# Methylphenidate-Induced Dyskinesia in a 47-Year-Old Female With Acute Lymphoblastic Leukemia

**DOI:** 10.7759/cureus.78995

**Published:** 2025-02-14

**Authors:** Olivia Vasey, Tyler Francisco, Eduardo D Espiridion

**Affiliations:** 1 Psychiatry, Drexel University College of Medicine, Philadelphia, USA; 2 Psychiatry, Tower Health Medical Group, West Reading, USA; 3 Psychiatry, Reading Hospital/Tower Health, West Reading, USA

**Keywords:** dyskinesia, methylphenidate, methylphenidate side effect, myelodysplastic syndrome, orofacial dyskinesia, stimulant

## Abstract

Dyskinesia describes a broad category of involuntary body movements. The presentation of dyskinesia is variable, ranging from quick jerking and twitching to smooth “dance-like” movements. Dyskinesia can occur in the extremities, face, or trunk. They can result from the use of certain medications and various medical conditions. One cause, explored in this paper, is dyskinesia as a side effect of methylphenidate use. We report on a 47-year-old woman with a past medical history of hypoplastic low-grade myelodysplastic syndrome with pancytopenia, acute lymphoblastic leukemia (in remission), and generalized fatigue treated with methylphenidate for her fatigue and decreased concentration. The patient presented from home to the emergency department with expressive aphasia, facial spasming, and involuntary extremity movement. Initial concern for stroke was ruled out and ultimately methylphenidate was determined to be the offending agent. Although our case focuses on an adult, cancer patient affected by dyskinesia following methylphenidate use, dyskinesia is a reported side effect with methylphenidate use in other populations. Clinicians should be aware of this association because of methylphenidate’s extensive use in the treatment of attention deficit hyperactivity disorder (ADHD) and its increasing use in other patient populations, like cancer patients and patients with brain injuries.

## Introduction

Methylphenidate (MTP) is a central nervous system (CNS) stimulant indicated in the treatment of attention deficit hyperactivity disorder (ADHD) and narcolepsy. MTP also has been used in off-label ways in clinical practice. It has also been useful in the treatment of patients with cancer, brain injury, and HIV. Though MTP’s complete mechanism of action is unknown, it is known to affect multiple neurotransmitters. Its main effects are thought to be due to inhibition of dopamine and norepinephrine reuptake. Dopamine receptor hypersensitivity and excess altered dopamine signaling, dysregulation of the other neurotransmitter systems, and genetic predisposition are some mechanisms that are thought to lead to dyskinesia [1]. This increases the neurotransmitter’s presence in the synapse, resulting in increased CNS stimulation.

Like other stimulant medications, MTP’s side effects can impact many body systems, including the CNS, gastrointestinal, and cardiovascular systems. Common side effects include growth retardation in children, insomnia, nervousness, restlessness, akathisia, headaches, nausea, vomiting, anorexia, weight loss, tachycardia, and hypertension [1,2]. Though reported less frequently, dyskinesia, angina, dysrhythmia, rash, and urticaria occasionally occur with MTP use [2]. Some of these side effects can severely impact the health of the patient and should be monitored for, especially with long-term use of the medication. Dyskinesia has been well-documented as a possible CNS side effect of MTP. Numerous case reports attribute orofacial and extremity dyskinesia and torticollis to its use [3]. Dyskinesia has been reported in ages ranging from toddlers [3] to the elderly [4] and with length of use ranging from one dose [5] to chronic use. Differential diagnoses for dyskinesia should be considered when evaluating these patients. Medication-induced dyskinesia has been associated with antiarrhythmics, anti-emetics, anti-epileptics, and psychotropic medications. Other causes of dyskinesia encompass a wide range of conditions, like Parkinson's disease, brain injury, Wilson’s disease, Tourette’s, stereotypies and tics, Huntington’s disease, tremors, myoclonus, spasmodic torticollis, athetosis, stroke, thyroid disease, multiple sclerosis, brain tumor, and restless leg syndrome [6]. We report an episode of acute facial and extremity dyskinesia with expressive aphasia in a patient receiving MTP for generalized fatigue due to acute lymphoblastic leukemia.

## Case presentation

A 47-year-old woman with a past medical history of hypoplastic low-grade myelodysplastic syndrome with pancytopenia, acute lymphoblastic leukemia (in remission), and generalized fatigue treated with MTP immediate-release tablet presented from home to the emergency department with expressive aphasia, facial spasming, and involuntary extremity movement.

The patient reported distressing somatic symptoms 30 minutes prior to arrival to the emergency department. She was at the grocery store when she began to experience abnormal spasms in her right lower extremity that prevented her from walking normally. She returned home, where her symptoms worsened and started to include her right upper extremity. Her husband promptly brought her to the emergency department. On arrival, she was having difficulty phonating, was unable to control bilateral extremities, primarily on the right side, and was having left facial movements/twitching. Later, the patient stated her extremities “were not listening to her” and “I couldn’t think of the right words to say.” These symptoms had never previously presented.

Neurology service was immediately consulted for initial concern of potential stroke given impaired speech, dystonia, and general ataxia (National Institutes of Health Stroke Scale (NIHSS) Score = 7). Computed tomography (CT) scan of the brain without contrast ruled out intracranial hemorrhage. CT angiography (CTA) and CT brain perfusion studies were completed, demonstrating no large vessel occlusion, no areas of hemodynamically significant stenosis, and normal CT perfusion. The patient was able to follow commands and answer questions appropriately. Given there was a bilateral involvement without losing consciousness, in addition to initial negative brain imaging, concern for stroke was reduced. The patient was deemed not a candidate for tissue plasminogen activator (tPa). She received 2 milligrams (mg) of intravenous (IV) benztropine without improvement in symptoms. She then received 4 mg IV lorazepam and symptoms began to improve. MTP was eventually held. MTP was not identified as the main culprit associated with the somatic symptoms, but it was held as it is a potential cause.

The patient was started on MTP-immediate release 5 mg daily two months prior to presentation by her oncologist for her low energy. It initially improved her fatigue, and she endorsed improved focus but noticed these effects would wear off by midday. Her oncologist increased her to MTP-extended release 18 mg daily two weeks prior to presentation. This provided additional benefits and would last all day long. The patient's outpatient oncologist has been managing her hypoplastic low-grade myelodysplastic syndrome with pancytopenia and acute lymphoblastic leukemia (in remission) with a current prescription of cyclosporine 100 mg BID. While a bone marrow transplant had been considered for pancytopenia, she was taking pegfilgrastim at the time of hospitalization; CBC with differential was ordered to establish baseline and observe trends (Table 1).

**Table 1:** CBC with differential RBC: red blood cell, MCV: mean corpuscular volume, MCH: mean corpuscular hemoglobin, MCHC: mean corpuscular hemoglobin concentration, RDW: red cell distribution width, MPV: mean platelet volume, ANC: absolute neutrophil count (H): Data are abnormally high, (L): Data are abnormally low, !: Data are abnormal

	Latest reference range & units	05/15/24, 18:53	05/16/24, 05:47	05/17/24, 07:16
White blood cell	4.8 - 10.8 x 10E3/µL	4.3 (L)	3.4 (L)	3.0 (L)
RBC	4.00 - 5.40 x 10E6/µL	3.41 (L)	3.24 (L)	3.36 (L)
Hemoglobin	12.0 - 16.0 g/dL	11.2 (L)	10.5 (L)	11.0 (L)
Hematocrit	35.0% - 47.0%	34.5 (L)	32.5 (L)	34.6 (L)
MCV	80.0 - 99.0 fL	101.2 (H)	10.3 (H)	103.0 (H)
MCH	27.0 - 34.0 pg	32.8	32.4	32.7
MCHC	31.0 - 37.0 g/dL	32.5	32.3	31.8
RDW	11.0% - 16.0%	16.2 (H)	16.4 (H)	16.2 (H)
Platelets	130 - 400 x 10E3/µL	71 (L)	66 (L)	59 (L)
MPV	8.0 - 13.0 fL	12.9	13.1 (H)	13.8 (H)
Segmented neutrophils	37% - 75%	37		22 (L)
Bands present	0% - 10%	2		2
Promyelocytes	0% - 1%	1		1
Myelocyte	0% - 1%	3 (H)		5 (H)
Metamyelocytes	0% - 1%	1		3 (H)
Lymphocytes	14% - 48%	30		36
Monocytes	0% - 12%	23 (H)		31 (H)
Basophils manual	0% - 2%	3 (H)		
ANC manual	2.00 - 8.00 x 10E3/UI	1.69 (L)		0.72 (L)
RBC morphology	Normal	Present !		Present !
Ovalocytes	None seen	Slight !		Slight !
Polychromasia	None seen	Slight !		Slight !
Anisocytosis	None seen	Slight !		Slight !
Macrocytes	None seen	Slight !		Slight !

**Figure 1:**
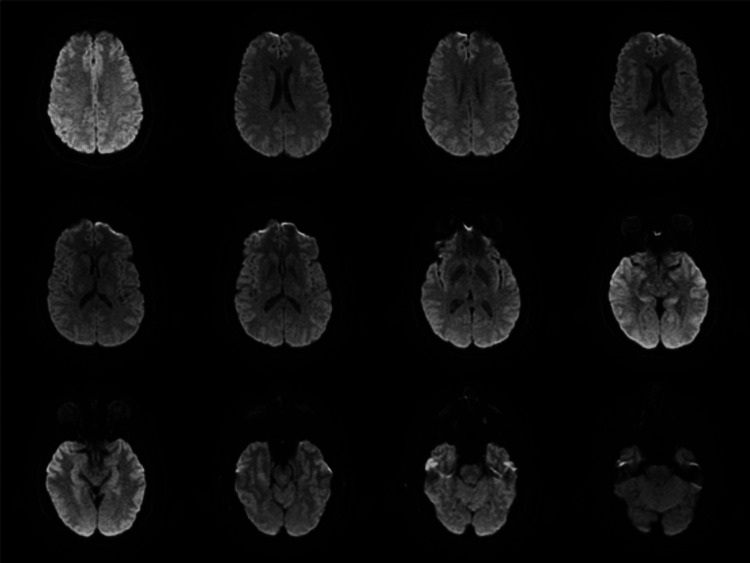
MRI brain without contrast Findings: The ventricular system is normal in size and configuration throughout. There is no hemorrhage, mass lesion, acute infarction or fluid collection. Normal signal flow voids are demonstrated within intracranial internal carotid and vertebrobasilar arteries. The sella, orbits, and visualized sinuses are grossly normal. Impression: No acute intracranial abnormality.

On day 2 of hospitalization, the patient reported improved but continued involuntary right arm movements that were not intentional. She also endorsed periodic right leg “tightening and pointing” and her face getting “stuck” for a few minutes. On exam, the patient’s right hand would alternate between pronation and supination in a “dance-like” movement that would slow and quicken throughout the evaluation. The patient also appeared to be grimacing at times as the left side of her face would contract and intermittently twitch. The patient reiterated that this was out of her control, and it was observed that symptom severity did not seem to fluctuate with patient distraction. The patient underwent brain magnetic resonance imaging (MRI) without contrast showing no acute intracranial abnormalities (Figure 1).

Based on improvement in symptoms with initial intervention, MTP was held while benztropine and lorazepam were administered. With a negative brain MRI, stroke was ruled out. It was recommended that the patient discontinue MTP upon discharge as it was determined to be the most likely issue because of her symptoms. While it is not inferred to be a definite cause of her somatic symptoms, the improvement of those symptoms occurred after the MTP was held. She was discharged with benztropine 1 mg by mouth every 12 hours as needed for dyskinesia and lorazepam 2 mg by mouth every 12 hours as needed if dyskinesia had not improved after at least 30 minutes after taking benztropine. Psychiatry recommended discussing bupropion with her treatment team after the resolution of dyskinesia to improve low energy and fatigue, considering symptoms as potentially secondary to underlying depression. Sleep hygiene education was also provided as the patient endorsed poor sleep most nights.

## Discussion

This case highlights a few aspects of MTP-related dyskinesia that have been reported in the literature and other aspects that have yet to be discussed comprehensively. Many case reports have been published concerning MTP use associated with dyskinesia in the context of ADHD treatment [5,7-11].

Still, no case reports have examined instances where the MTP was used to treat cancer-related fatigue. In this particular patient, her immediate-release MTP was improving her low energy levels and focus, but only until mid-day. Increasing her dose from 5 mg to 18 mg and switching to extended-release helped lengthen the duration of benefits. The use of MTP in cancer patients is, however, controversial. Some studies did find that “MTP as needed was significantly more effective than placebo in relieving fatigue after two and five hours” in patients with cancer [12]. Meanwhile, other studies found MTP to not be more effective than placebo, with fatigue stabilizing within six days of starting both the placebo and MTP [13]. Individualized decisions must therefore be made weighing the possible risks and benefits of prescribing MTP in this population while monitoring the patient for side effects and efficacy of treatment. Though no studies have been done to understand the relationship between MTP and dyskinesia in the population of patients utilizing the medication for cancer-related fatigue, there have been multiple studies linking MTP use with dyskinesia in patients with ADHD. One such study from 2011 found that there was a significant difference between the baseline total Abnormal Involuntary Movement Scale (AIMS) score in the ADHD and the control groups, with the ADHD subjects evidencing substantially higher severity than controls [14]. Another study looked at the differences in AIMS scores between three groups. A control group, an ADHD group treated with MTP, and an ADHD group that was treatment-naive. The study found that the treated ADHD group had significantly higher AIMS scores than either of the other groups both before and 1.5 hours after the administration of MTP [15]. This patient’s dyskinesia symptoms resemble symptoms found in other case reports. Orofacial dyskinesia is a common manifestation of MTP-associated dyskinesias. Presentations of orofacial dyskinesias in other case reports included oromandibular dystonia in an 18-year-old patient being treated for ADHD [6], isolated orofacial dyskinesia in a six-year-old female [8], and isolated tongue rolling movements in a seven-year-old female [9]. Extremity dyskinesia can also occur as a side effect of MTP, as it did in the patient described in our report. Acute limb dyskinesia has been described in cases with orofacial dyskinesia in adolescents being treated for ADHD [5,10,11]. The literature contains few reports of dyskinesia in adults treated with MTP, likely due to its main use for ADHD in adolescents. However, several case reports have noted this side effect in the adult and elderly populations. One such paper identified an 87-year-old Parkinson’s patient who developed significant choreoathetosis after only two doses of MTP [4]. The choreoathetosis subsided with the discontinuation of MTP. Another paper reported dyskinesias in two adult patients using MTP as an adjuvant therapy following stroke [16]. A review of the literature has shown that MTP-associated dyskinesia occurs across many ages and with varying presentations.

## Conclusions

Although our case focuses on an adult, cancer patient affected by dyskinesia following MTP use, dyskinesia is a reported side effect with MTP use in other populations. It is a possible effect in all age ranges, in patients with various indications for treatment, and with as little as one dose of the medication. The dyskinesia experienced can also differ in severity and body parts affected. In this patient, dyskinesia developed after prolonged use and a recent dose increase of MTP and affected both her extremities and face to a degree severe enough to warrant a visit to the emergency department. Based on the severity of symptoms, patients may present with dyskinesia to emergency departments, their primary care providers (PCPs), or specialists. It is important for physicians to be able to identify dyskinesia and know of its association with specific medications. In most cases, discontinuation of the medication resolved the symptoms. It is important to note that dyskinesia is a rare side effect of MTP use and that it is a generally safe drug to use for the treatment of ADHD and cancer-related fatigue. However, clinicians prescribing MTP should be aware of this association.
